# Inevitable Loss and Prolonged Grief in Police Work: An Unexplored Topic

**DOI:** 10.3389/fpsyg.2020.01178

**Published:** 2020-05-29

**Authors:** Konstantinos Papazoglou, Daniel M. Blumberg, Peter I. Collins, Michael D. Schlosser, George A. Bonanno

**Affiliations:** ^1^Yale School of Medicine, New Haven, CT, United States; ^2^California School of Professional Psychology at Alliant International University, San Diego, CA, United States; ^3^Division of Forensic Psychiatry, University of Toronto, Toronto, ON, Canada; ^4^Threat & Behavioural Assessment Team, Criminal Behaviour Analysis Section, Ontario Provincial Police, Orillia, ON, Canada; ^5^Police Training Institute of Illinois, University of Illinois at Urbana-Champaign, Champaign, IL, United States; ^6^Teachers College, Columbia University of New York, New York, NY, United States

**Keywords:** death, loss, prolonged grief, bereavement, law enforcement, health, wellbeing, performance

## Abstract

The present manuscript presents foundational constructs related to death and loss (i.e., grief, bereavement, prolonged grief) providing empirical findings from recent research on the impact of death and loss on police officers’ health, behavior, and overall functioning. Police officers are routinely exposed to death. In many instances, officers’ contact with decedents includes, among others, victims of accidents, catastrophes, or violent crimes and witnessing the intense emotional suffering of relatives of the deceased. Additionally, it is not uncommon for officers to experience the loss of fellow officers from on-duty deaths and permanent, career-ending injuries. Simultaneously, like everyone, police officers have to cope with deaths of loved ones in their personal lives. The result is that officers’ health and well-being are likely compromised because of the systematic exposure to on- and off-duty deaths. In this perspective paper, death and loss in law enforcement are explored in an attempt to raise awareness and increase attention to this area of police work. In addition, the authors list a number of prophylactic intervention strategies that would support officers cope with the impact of loss and death and promote their own resilience.

## Introduction

Death is an essential, if unfortunate, fact of life for all species on this planet, and, as one ages, the probability of being exposed to the loss of a loved one becomes greater (Bonanno et al., 2012). To deal with this reality, humans have developed a variety of death-related rituals, which vary across cultures (Bonanno, 2009). Bereavement is commonly defined as the painful experience of losing a loved one, while grief pertains to the spectrum of emotional, psychophysiological and symptomatic responses to that loss over time (Shear, 2012, 2015; Shear et al., 2013). Perhaps a better understanding of these phenomena can be obtained by using a biological analogy wherein bereavement corresponds to an injury, and grief corresponds to the painful inflammatory response to it. Although responses to the death of a loved are heterogeneous, most people experience acute grief, which manifests in the form of strong yearning, longing, sadness, stress, and intrusive thoughts and images of the deceased one. These reactions can last from a few weeks to months or even years. To deal with grief, individuals engage in mourning, which consists of the rituals and psychological processes that help them adjust to a world without their loved one (Shear, 2012, 2015). At the end of a successful mourning period, the person is usually able to return to the baseline (before experiencing grief) level of functioning. Nonetheless, many people grieve briefly, most intensely within a few weeks, while others grief for longer periods of time, and some suffer prolonged reactions.

In the present paper, the discussion of loss focuses on the experience of death and grief among police officers. After describing recent research on the phenomenon in the general population, we focus on the unique challenges faced by police officers who are exposed to death on a regular basis. Beyond a description of these challenges, we introduce a variety of coping mechanisms that have proven helpful to police officers who experience grief. Additionally, preventative measures are presented, which, when utilized by police officers, can serve to inoculate officers from some of the more deleterious grief reactions.

## Prototypical Grief Outcomes

Longitudinal studies of grief reactions have consistently identified three prototypical patterns (Bonanno et al., 2012). The majority of bereaved individuals are able to return to their normal level of functioning relatively soon after a loss and over time show a stable trajectory of mental and physical health or *resilience*. Others show a more prolonged *recovery* trajectory characterized by elevated grief and distress that gradually abates over the course of the first year or two after the loss. Finally, a small but important minority of bereaved individuals experiences *prolonged grief* that endures for at least several years and usually longer.

Prolonged grief is an unusually severe condition that impairs a person’s ability to function in important domains in life. It is estimated that approximately 10–15% of people who lose a loved one experience prolonged grief (Lundorff et al., 2017), particularly after the death of a romantic partner or child and in cases of sudden or violent death (e.g., accidents, homicide, suicide) (Nakajima et al., 2012; Burke and Neimeyer, 2014; Shear, 2015). Based on the American Psychiatric Association (APA) updates on proposed changes to DSM-5 (American Psychiatric Association [APA], 2013, 2020), prolonged grief is recommended to be added as a new diagnosis considering recent research outcomes in this condition. More specifically, the proposed diagnostic criteria for the prolonged grief disorder are (American Psychiatric Association [APA], 2020): (A) The death of a person closed to the bereaved at least 12 months previously, (B) Since death a grief response that is characterized yearning/longing or pre-occupation with thoughts or memories for the deceased person nearly every day for at least the last month, (C) Three of the following symptoms experienced to a clinically significant degree nearly every day for at least the last month: identify disruption, marked sense of disbelief about the death, avoidance of reminders of the deceased person, intense emotional pain, difficulty moving on with life, emotional numbness, feeling that life is meaningless, and intense loneliness, (D) The disturbance causes clinically significant distress or impairment in social, occupational, or other important areas of functioning, and (E) The duration of those symptoms should exceed the expected cultural, social, or religious norms for the individual’s culture. Shear (2012), who has been one of the proponents for the aforementioned addition of prolonged grief as a new DSM-5 diagnosis, contends that such types of loss force individuals to confront not only the death of their loved one, but also the fact of their own mortality. Symptoms of prolonged grief may include: intense yearning; longing; emotional pain; frequent, pre-occupying thoughts and memories of the deceased; inability to accept the loss; self-identity confusion; emotional numbness; loss of trust; sense of shock; bitterness; and the inability to imagine a meaningful future without the deceased person (Boerner and Schulz, 2009; Bullock and Bonanno, 2013; Maccallum et al., 2017). In addition, Burke and Neimeyer (2014) note that prolonged Grief may have a spiritual impact, as it can lead individuals to feel distant from God or some other type of Higher Power, which can in turn cause them to isolate themselves from other members of their spiritual or religious community. Returning to the above biological analogy, prolonged grief can be thought of as an infection that develops in a wound.

### Predictors of Grief Outcomes

Bereaved individuals showing the resilient trajectory of minimal grief have been found to exhibit a number of key protective factors. Resilient individuals who had lost a spouse were observed to be more emotionally stable and extraverted, less lonely, more likely to perceive others as being available to listen to their concerns, and have a greater capacity to experience comfort when recalling memories of the deceased (Mancini et al., 2015). Studies measuring facial expressions of emotion during bereavement have documented that bereaved individuals who are able to utilize positive emotions early after a loss are more likely to show reduced grief and depression at later points in bereavement (Bonanno and Keltner, 1997; Keltner and Bonanno, 1997).

A prospective study that tracked married individuals in the years before and after the death of their spouse found that those who reported greater instrumental support (i.e., availability of help with the necessities of daily living), acceptance of death, and belief in a just world while married were more likely to show a stable, resilient outcome during bereavement (Bonanno et al., 2002).

In an experimental study comparing individuals with symptomatic and asymptomatic prolonged grief, Gupta and Bonanno (2011) found that those who suffered from prolonged grief showed less flexibility when asked to modulate the expression of emotions. These findings further indicate that prolonged grief can lead to maladaptive emotional regulation, which may disrupt social relationships and decrease levels of well-being. Indeed, the enhancement of negative emotions can be problematic when someone expresses anger in a context in which they expect to build bonds of trust and rapport. Similarly, emotion suppression among prolonged grief sufferers may result from experiencing more negative than positive emotions, which can be detrimental to one’s well-being and social relationships.

In a related bereavement study, Bullock and Bonanno (2013) measured attentional biased among bereaved participants suffering from prolonged grief, bereaved participants who were asymptomatic, and married individuals as they viewed facial expressions of emotions. Notably, those in the prolonged grief group exhibited a general attentional bias away from happy faces and, when primed with the name of their spouse, away from sad faces. Diminich and Bonanno (2014) observed similar results, finding that prolonged grief sufferers lacked the ability to facially express positive and negative emotions, thus highlighting the presence of emotional dysregulation and disrupted emotional processes. Attention bias was also observed among prolonged grief sufferers during an emotional Stroop task in which participants were presented with death-related and neutral cue words (Maccallum and Bryant, 2010). Participants who experienced prolonged grief were slower to respond to death-related words compared to asymptomatic bereaved participants. These results led the researchers to theorize that death-related words trigger rumination, negative mood states, and distressing memories that then slow the response to death-related words by increasing attentional bias.

Another recent study, Schneck et al. (2019) examined the Emotional Stroop and other tasks while bereaved individuals were in an fMRI scanner. Using machine learning, they first identified the neural signature for brief instances when participants’ attention was momentary diverted by the unexpected appearance of the deceased love one’s name. Next, they identified instances when this neural signature emerges as participants engaged in a subsequent cognitive task and periodically probed participants about their thought processes. Severely grieving participants tended to show the neural signature associated with the deceased’s name deceased during the cognitive task, and also report conscious thoughts of the deceased during that task. By contrast, when bereaved participants with minimal grief showed the neural signature for the deceased, they tended not to report thinking about the deceased. Instead, they were more likely to show neural activity commensurate with through suppression.

### Prolonged Grief vs. Post-traumatic Stress Disorder

Although prolonged grief and post-traumatic stress disorder (PTSD) have distinct symptoms, these symptoms overlap in certain instances (Nakajima et al., 2012; Shear, 2012; Malgaroli et al., 2018). These two afflictions are distinct in that fear, terror, and anxiety are the dominant symptoms of PTSD, while sadness and yearning tend to be the most prominent symptoms of prolonged grief. In addition, whereas intrusive thoughts and symptoms are related to the traumatic incident in PTSD, they are linked to the deceased person in prolonged grief. Similarly, individuals with PTSD avoid places, activities, or individuals associated with the traumatic incident, while prolonged grief sufferers avoid feelings, places, and activities that remind them of the deceased. Furthermore, individuals with PTSD suffer from hypervigilance in relation to threat- or trauma-related cues; in contrast, those with prolonged grief may experience physiological dysregulation due to disengagement or disconnectedness from interpersonal contact. It should be noted that both PTSD and prolonged grief can co-occur in cases involving the sudden, unexpected, violent death of a loved one (Nakajima et al., 2012). For instance, police interviews, forensic investigations, attorney interviews, and testimony in court may exacerbate the symptoms of PTSD and prolonged grief for individuals who have experienced this type of loss (Wenzel, 2002; Gibson, 2008). In addition, co-morbid conditions such as depression may also exacerbate symptoms of PTSD or prolonged grief.

Of all mental health conditions experienced by police officers, PTSD has received the most clinical and research attention (Gersons et al., 2000; Difede et al., 2007). In addition, PTSD and prolonged grief are often comorbid as shown by a growing body of research (e.g., Wenzel, 2002; Gibson, 2008; Nakajima et al., 2012); however, it appears that PTSD symptom reduction may be more challenging in the treatment context when such comorbidity exists (Smid et al., 2018). Despite the treatment challenge when PTSD and prolonged grief are comorbid, PTSD evidence-based treatment (e.g., Cognitive-Behavioral Therapy, Exposure Therapy) has been shown to be significantly efficacious in PTSD symptom improvement among first responders who suffer from PTSD (Gersons et al., 2000; Difede et al., 2007). Most police officers may not ultimately be diagnosed with PTSD, however, most officers experience loss at some point during their law enforcement careers. Nevertheless, especially in the context of specialized police mental health services with a focus on PTSD, specific attention should be emphasized to the mental health effects of losses of loved ones on police officers’ mental health and well-being (Smid et al., 2018).

## Loss in Police Work

Imminent danger is endemic to police work. As such, recruits are instructed about personal and civilian safety as well as survival on the streets from the moment they join the police academy. During training, officers and cadets are consistently reminded that a critical aspect of surviving on the streets is to recognize the fact that almost every encounter has the potential to become life threatening. In addition, the array of equipment used by police officers—from body armor, to handguns, to Tasers, to the bullet-proof windows in police cruisers—also serve as reminders, either explicitly or implicitly, of the potential risks inherent to the profession. Of course, no officer wakes up in the morning with the intention of shooting someone or even drawing their firearm; however, they are trained to be prepared for any level of risk their work may entail (Carson, 2014).

In their article on the environment of death and its influence on police officers, Sugimoto and Oltjenbruns (2001) catalog some of the death-related events that Sugimoto experienced as an officer, including: a girl who was hit by a vehicle while she was snow-sledding (the vehicle had no way of avoiding her) and died on the scene as a result of massive head trauma; a man who was dismembered as a result of being run over by a train, with officers being forced to locate his severed body parts in order to identify the deceased; an officer who witnessed Satanists releasing a dog that had been skinned alive to run around a cemetery, and many other incidents included in the initial list but not reported in this manuscript by the authors for the sake of brevity.

Readers should be reminded that the above list of death-related incidents is not exhaustive. In addition, it should be noted that these death-related scenarios are not restricted to those in which officers’ lives are jeopardized; rather, they encompass any scenario wherein officers must respond to calls that may expose them to danger or death. For example, in the aftermath of the 9/11 terrorist attacks police officers rushed to ground zero in an attempt to save civilians, even though they were well aware that the buildings were going collapse and that they would likely die. This sort of selfless behavior can also be observed in other critical incidents, for example, active shootings. Public and social media feature a plethora of images of officers approaching an active shooter’s location, while civilians run to escape (expectedly and understandably). Given the frequency of these death-related scenarios across police departments, it is highly likely that interviews with other officers would produce a list that is similar to the one documented by Sugimoto and Oltjenbruns (2001).

In addition to encountering death on a regular basis on-duty, police officers, like everyone else, have to confront loss in their personal lives. Sometimes this involves the death of a co-worker from an illness or suicide, which blurs the line between professional and personal life. It is not uncommon, however, for police officers to tackle the emotional burden of grief from the loss of a loved one. Although some of these deaths follow protracted illnesses, in some cases, this loss occurs from an incident that is remarkably similar to events that the officers face on-duty (e.g., a fatal traffic accident). In such instances, again, the separation between personal and professional life is extremely difficult to maintain, and officers find it difficult to grieve the loss of their loved one while attempting to continue working effectively. For example, an officer was faced with quitting if his agency did not provide him with an alternative work assignment after the suicide of his 16-year old daughter, because he was no longer emotionally able to work a patrol assignment where he routinely was called to the scene of suicides.

### The Complexity of Loss in Police Work

As previously discussed, police officers are exposed to myriad incidents involving death or that have a high risk of becoming deadly. Moreover, a death-related situation in police work is often direct (e.g., an officer or a civilian gets shot in the line of duty, dead body at a crime scene, etc.) and prolonged. For instance, patrol officers responding to a violent crime scene have to secure the perimeter with their colleagues and wait until the coroner and detectives arrive. Officers in such situations can be expected to feel negative emotions such as anger and agitation toward the perpetrator(s), especially when the victims are minors or elders, as the officer may identify them with members of their own family (e.g., children, parents).

Police work is characterized by an atmosphere that can be described as “death saturated,” which officers are exposed to either directly or indirectly, and often in a prolonged manner. Direct exposure to this death-saturated atmosphere occurs in officers’ responses to crime scenes, the reports they write afterward, and providing testimony in criminal trials related to these cases. On the other hand, officers can be indirectly exposed to death in a number of ways. For example, the main lobby of every police precinct and police academy contains a memorial featuring the images of police officers who have sacrificed their lives in the line of duty. Even though these memorials are intended to honor the heroic sacrifice made by these officers (and rightly so!), they also consciously or unconsciously remind other officers that death is a fact of life in police work, and that they could very well be next. Furthermore, as noted above, the potential danger and unpredictability of police work is reflected in the emphasis placed on survival in police training, as well as in the equipment that officers carry during their shifts. Henry (1995), a psychologist and former sergeant with the New York Police Department, accurately illustrates this ever-present danger this dynamic is a constant and pervasive sense of distrust, skepticism, and suspicion, born out of the recognition that even the seemingly most ordinary and mundane event can ultimately have a deadly outcome….that one may be called upon to use deadly force under highly ambiguous circumstances.” (p. 94).

The following passage relates the experience of a veteran officer, who successfully neutralized an active mass shooter. This officer was interviewed by the first author of this manuscript as part of a research project named, “Listening to Their Voices of Bravery and Heroism” (Papazoglou, 2016):

I’ve seen a lot of death by gunshots and they, it just tears apart, it shreds your face or your chest or your arms or legs. And that is shocking to see, but not, not when you see it all the time…but when we see it all the time, it’s not shocking anymore. But for the average person…they’re not going to have a clue. No matter how descriptive you are, they’re not going to get it…but it’s almost like you have to see the most graphic, violent deaths in order to understand what the cop or the soldier had to do. It’s just about that extreme, otherwise they’re having, they are going to have no clue.

In addition, if some of the pioneering research on grief and loss, reviewed above, is applied to law enforcement, it becomes clear that prolonged grief can have an egregious effect on police performance. It is critical that officers are able to regulate their emotions when interacting with civilians or attempting to de-escalate critical situations, and emotional dysregulation, attentional bias, or the inability to express positive or negative emotions may significantly impair their ability to do so, which can have dire consequences if the use of force is required. Moreover, emotional dysregulation has broader implications, as it can also affect officers’ relationships with their colleagues, family members, and friends.

### Police Coping Mechanisms in the Light of Death or Loss

The following list outlines a number of the ways in which officers cope with exposure to death or loss in the line of duty:

• Officers will frequently use comical or sarcastic terms to minimize or trivialize the seriousness of a case. Henry (1995) cites the following informal terms that police often use when referring to Dead on Arrivals (DOAs): “fresh” (recent death), “ripe” (noticeably decomposing body), “floater” (body in an advanced decomposition state), “jelly belly” (extremely swollen corpse), “road pizza” (a corpse mutilated by a motor vehicle accident), and “two canner” (burned body).
• When an officer is killed in the line of duty, off-duty officers will pin their shields to their civilian clothes while attending the wake (Henry, 2004) as a way of displaying their solidarity and mourning their fallen blue brother or sister.
• Officers are usually in charge of keeping the public and media away from the crime scene, particularly for violent crimes. In contrast, detectives are often tasked with providing the media with updates and information about a crime, as they are the ones who are in charge of gathering and analyzing the crime scene and any evidence collected. Unfortunately, the frequency with which detectives are exposed to violent crime and their sense of duty in attempting to catch those responsible can desensitize them and lead them to treat violent crime as routine cases (i.e., a regular job) (Henry, 2004).
• When officers lose a colleague, it is common for them to gather and tell stories about their fallen brother or sister. This is often aided by with the mild or moderate use of alcohol, as it helps to “oil the mechanism of self-expression in a supportive atmosphere” (Miller, 2007, p. 14). To this end, in a large sample of police officers (*n* = 747) from large metropolitan areas, Ballenger et al. (2011) found that both male and female officers reported adverse lifetime consequences from alcohol such as general psychiatric symptoms, adverse social and interpersonal consequences, and occupational stress.

### The Impact of Loss and Death on Officers

The experience of loss and death may negatively impact officers’ lives and performance in multiple ways. It is not uncommon for officers to feel anger and irritability toward the perpetrators of violent crimes, and officers who experience the loss of a colleague or civilian may experience survivor’s guilt. These responses may lead officers to act impulsively in order to satisfy the overwhelming urge to act and do something (Miller, 2007). In contrast, officers may actively avoid places, people, or situations related to the source of grief. Similarly, officers may be inundated by images and thoughts of a death-related situation, which can significantly impair their job performance and even put their career in jeopardy. As discussed in previous sections, officers may experience grief and trauma-related reactions in response to cases of traumatic death (e.g., death of a partner). In addition, such experiences may force officers to confront their own mortality, which can lead to a sense of existential angst (Gibbs et al., 2014). Moreover, one study, which drew upon data from the New Jersey Cop 2 Cop hotline, found substantially higher numbers of calls relating to suicide ideation and urgent care from police officers who worked in the proximity of the 9/11 (Violanti et al., 2006). Other studies have also found that exposure to deadly shooting situations increases officers’ likelihood of experiencing suicidal thoughts and behaviors (Stanley et al., 2016). The following statement was provided by a veteran sergeant with a Canadian police force, and it details their experience with a shooting incident and their subsequent struggle with suicidal ideation and behaviors. This officer was interviewed by first author of this manuscript as part of a research project entitled, “Listening to Their Voices of Bravery and Heroism” (Papazoglou, 2016):

I had driven myself to a point where I felt that this moment was overwhelming and maybe it would be easier if I just left and killed myself. So I went to the basement to plan out my suicide and I had great sense of relief as I had in the cell, it was this great sense of relief of I finally figured out this is going to work well for me. My wife went to work the next day and she always took the kids with her …. and I was sitting in the living room and I remember thinking “ok revisit this again” and so I went down to the basement to revisit the whole plan to make sure I thought things through and the doorbell rang and I ignored it, it was RS, RS had been an officer involved in the shooting who had talked to me several times and was a friend of mine….so I finally came out of the basement and answered the door and he was “what are you doing” and I said “I can’t. I can’t keep going, that’s enough” and he said “stop, stop talking like that, that’s not right” and we sat down and we chatted and we chatted for several hours I think and finally, my wife came home and I was okay and I was able to, I told her and then we, she took a couple of days off to stay with me and we worked through it and I realized then that in spite of everything being in place, that’s how it can happen still.

### Police vs. Civilian Loss: What Is Missing?

As we discussed earlier, the scholarly literature on grief and bereavement identifies the loss of a loved one as a necessary component for the experience of prolonged grief. However, aside from the loss of a partner or family member, this component is missing in police work. Nevertheless, there is an apparent gap in the literature regarding the conceptualization of the complexity of the nature of death and loss experienced by law enforcement. Does this mean that officers do not experience Prolonged grief? Do they experience a type of prolonged grief that is both distinct from and overlaps with the sort of prolonged grief that is experienced by civilians? The above discussion of the complexity of loss in policing produces a key idea: the experience of death and loss appears to occur accumulate in a spiral pattern throughout an officer’s years of service.

Although some articles discuss the experience of death and loss in policing, researchers have yet to study of how officers experience and cope with death, loss, and grief. In addition, it is important for researchers to further explore whether or not officers experience prolonged grief, and how this prolonged grief is similar or distinct from that experienced by civilians. Furthermore, it is critical to develop a more robust diagnostic approach that accounts for the fact that police officers can suffer from prolonged grief, even if they have not lost a loved one. All of these areas represent important directions for future research by clinical practitioners and researchers.

### Action Plan: Prophylactic Intervention

• Mindfulness exercises provides officers with a non-judgmental method of self-observing and reflecting upon how their body reacts to death-related situations. This is crucial as it prevents officers from any catastrophic judgments and negative emotionality (Shear, 2012; Chopko et al., 2018).
• Imagery exercises can help officers create a serene space where they can maintain a zone of calmness and safety (Shear, 2012; Manzella and Papazoglou, 2014).
• Gratitude letters can help officers appreciate the value of their services in death- or loss-related incidents. When practiced regularly, this technique has been shown to benefit officers, especially in terms of helping them to feel satisfied and appreciative for helping those who suffer and maintaining community welfare (Toepfer and Walker, 2009; Papazoglou and Andersen, 2015; Papazoglou et al., 2020).
• Psychoeducation about loss and death in policing should be provided to officers and their families by health care professionals, as it will enable them to understand which reactions to death- or loss-related situations are to be expected. In addition, these healthcare practitioners can provide science-based information and realistic reassurance to officers and their families about grief and bereavement in policing (Miller, 2007). Furthermore, psychoeducation can help officers and their families understand that time may not heal their police-work-relate grief, and that therapy may be integral to this process.
• Mental health clinicians could conduct periodic assessments in order to identify officers who may be experiencing, or who may be at high risk for, prolonged grief symptoms as a result of exposure to critical incidents in the line of duty. Currently, some police departments may already employ periodic psychological assessments as part of their policy; however, such assessments are more likely focused on PTSD or other trauma-related symptoms (Trottier and Brown, 1995; Papazoglou, 2017). The authors suggest that such assessments also be modified to incorporated prolonged grief reactions or symptoms officers may experience. Identified officers can then be provided with prolonged grief clinical treatment to overcome any potential challenges, which will help to ensure that they are able to continue to perform their duties efficiently.
• The role of peer support groups is also integral in supporting officers’ dealing with trauma and loss-related reactions; as part of those groups, officers feel that they are in a safe context surrounded by peers who are willing to listen to their concerns and provide them with empathic feedback. In addition, peer support groups may utilize a variety of strategies such as humor to provide support to officers who deal with trauma or/and loss-related reactions (Evans et al., 2013).
• Spiritual and religious practice has been shown to be quite supportive for police officers especially during times of high distress (Chopko et al., 2016). To this end the role of police chaplains and other spiritual or religious leaders is vital in helping officers elaborate and explore on any existential or spiritual/religious concerns raised by exposure to death and loss in general (Charles et al., 2014).
• The literature indicates that officers who are single with no children are more likely to be exposed to death-related incidents due to thrill-seeking behavior (Kachurik et al., 2013). In addition, these officers tend to spend more time at work than their married peers, who generally prioritize spending time with their families. Furthermore, more experienced officers are less likely to be affected by death-related incidents or high-risk situations because they are more discreet in exercising caution during risky situations than their less experienced peers (Kachurik et al., 2013). As such, grief-related assessment should focus on single, childless officers who are relatively inexperienced, as this group has the highest risk of suffering as a result of death- or loss-related incidents.
• Similar to Bonanno et al. (2012) recommendations on resilience promotion in relation to potentially traumatic incidents, police training and clinical practice should focus on targeted behaviors among those who are most likely to instill adaptation to death- or loss-related incidents. In addition, officers who appear to have a high risk of suffering due to exposure to death- or loss-related incidents should be assessed and provided with interventions tailored to meet their needs (Bonanno et al., 2012).

## Closing Thoughts

The present paper described the ubiquitous role of death and loss in police work. For as much as most police officers enter the profession with the desire to help people, their jobs regularly expose them to human tragedies. These situations leave officers in a perpetual state of powerlessness. Coupled with deaths that occur among friends, family, and coworkers, police officers are at great risk of prolonged grief reactions. Therefore, it is of significant importance for police executives to recognize the problem and begin to provide more than post-incident resources. Police officers will benefit from preventative measures designed to boost resilience and to strengthen their spiritual foundation. Although exposure to death and loss are inevitable for police officers, there is much that can be done to thwart excessive and unnecessary prolonged grief reactions.

## Author Contributions

KP, DB, PC, MS, and GB: conceptualization, methodology, and writing–review and editing. N/A: software, validation, formal analysis, data curation, and funding acquisition. KP, PC, and DB: investigation and writing–original draft preparation. KP, PC, DB, and GB: resources. KP and DB: visualization. KP: supervision and project administration.

## Conflict of Interest

The authors declare that the research was conducted in the absence of any commercial or financial relationships that could be construed as a potential conflict of interest.
